# Protective anti-chlamydial vaccine regimen-induced CD4^+^ T cell response mediates early inhibition of pathogenic CD8^+^ T cell response following genital challenge

**DOI:** 10.1093/femspd/ftae008

**Published:** 2024-04-29

**Authors:** Ashlesh K Murthy, Erika Wright-McAfee, Katerina Warda, Lindsay N Moy, Nhi Bui, Tarakarama Musunuri, Srikanth Manam, Clemence Z Chako, Kyle H Ramsey, Weidang Li

**Affiliations:** College of Veterinary Medicine, Midwestern University, Glendale, AZ 85308, USA; College of Veterinary Medicine, Midwestern University, Glendale, AZ 85308, USA; Arizona College of Osteopathic Medicine, Midwestern University, Glendale, AZ 85308, USA; Arizona College of Osteopathic Medicine, Midwestern University, Glendale, AZ 85308, USA; Arizona College of Osteopathic Medicine, Midwestern University, Glendale, AZ 85308, USA; Arizona College of Osteopathic Medicine, Midwestern University, Glendale, AZ 85308, USA; College of Veterinary Medicine, Midwestern University, Glendale, AZ 85308, USA; College of Veterinary Medicine, Midwestern University, Glendale, AZ 85308, USA; College of Veterinary Medicine, Midwestern University, Glendale, AZ 85308, USA; College of Graduate Studies, Midwestern University, Glendale, AZ 85308, USA; College of Veterinary Medicine, Midwestern University, Glendale, AZ 85308, USA

**Keywords:** Chlamydia, CD4^+^ T cells, protective immunity, CD8^+^ T cells, reproductive pathology

## Abstract

We have demonstrated previously that TNF-α-producing CD8^+^ T cells mediate chlamydial pathogenesis, likely in an antigen (Ag)-specific fashion. Here we hypothesize that inhibition of Ag-specific CD8^+^ T cell response after immunization and/or challenge would correlate with protection against oviduct pathology induced by a protective vaccine regimen. Intranasal (i.n.) live chlamydial elementary body (EB), intramuscular (i.m.) live EB, or i.n. irrelevant antigen, bovine serum albumin (BSA), immunized animals induced near-total protection, 50% protection, or no protection, respectively against oviduct pathology following i.vag. *C. muridarum* challenge. In these models, we evaluated Ag-specific CD8^+^ T cell cytokine response at various time-periods after immunization or challenge. The results show protective efficacy of vaccine regimens correlated with reduction of Ag-specific CD8^+^ T cell TNF-α responses following i.vag. chlamydial challenge, not after immunization. Depletion of CD4^+^ T cells abrogated, whereas adoptive transfer of Ag-specific CD4^+^ T cells induced the significant reduction of Ag-specific CD8^+^ T cell TNF-α response after chlamydial challenge. In conclusion, protective anti-chlamydial vaccine regimens induce Ag-specific CD4^+^ T cell response that mediate early inhibition of pathogenic CD8^+^ T cell response following challenge and may serve as a predictive biomarker of protection against *Chlamydia* -induced chronic pathologies.

## Teaser

Protective anti-chlamydial vaccine regimens induce Ag-specific CD4^+^ T cell response that mediates early inhibition of pathogenic CD8^+^ T cell response following challenge and may serve as a predictive biomarker of protection against Chlamydia -induced chronic pathologies.

## Introduction

*Chlamydia trachomatis* is the leading cause of sexually transmitted bacterial disease worldwide. Significant efforts are being made to develop a vaccine to prevent female reproductive pathology. We and others have demonstrated previously that IFN-γ-producing Ag-specific CD4^+^ T cell response induced by vaccination regimens is necessary, and a sufficient source of IFN-γ production, to induce protection against reproductive pathology in naïve recipient mice that are challenged with *C. muridarum* (Morrison and Morrison 2001, Li et al. 2008, Gondek et al. 2012). We and others have also identified previously a number of chlamydial antigens as putative vaccine candidates (Pal et al. 2001, Murthy et al. 2007, Murthy et al. 2011, Yu et al. 2012, Yu et al. 2016). Whereas immunization with many chlamydial antigens induces a robust Th1 Ag-specific CD4^+^ T cell response, only a subset of regimens induces early resolution of infection and/or significant reduction of chronic reproductive tract pathology (Yu et al. 2016). Furthermore, multi-functional CD4^+^ T cells that produce TNF-α along with IFN-γ have been shown to correlate better with early reduction in bacterial shedding when compared to regimens that induce predominantly IFN-γ-producing CD4^+^ T cells (Yu et al. 2016). However, regimens that induce early reduction of bacterial shedding do not always induce reduction of chronic reproductive tract pathology, and conversely some regimens that significantly reduce chronic reproductive tract pathology do not induce early reduction of chlamydial shedding (Andrew et al. 2013, O'Meara et al. 2014). As such, immune correlates that accurately predict protective immunity against upper reproductive pathology, the prime reason that necessitates an anti-*Chlamydia* vaccine, have yet to be defined.

To this end, we have demonstrated previously that TNF-α-producing CD8^+^ T cells cause chlamydial reproductive pathologies (Murthy et al. 2011). In addition, TNF receptor 2 on CD8^+^ T cells and TNF receptor 1 on non-CD8^+^ T cells contribute significantly to chlamydial pathogenesis (Manam et al. 2015). Importantly, CD8^+^ T cells with specificities to non-chlamydial antigens do not induce reproductive pathology following genital chlamydial infection (Manam et al. 2013, Vlcek et al. 2016), suggesting that antigen (Ag)-specific CD8^+^ T cells mediate chlamydial pathogenesis. As such, we hypothesized that the reduction/inhibition of Ag-specific CD8^+^ T cell TNF-α response after immunization and/or challenge would correlate with the protective efficacy of a vaccine regimen against chronic reproductive pathology. In addition to TNF-α response, we evaluated the production of two other cytokines as activation markers of Ag-specific CD8^+^ T cells, IFN-γ and interleukin-17 (IL-17). Furthermore, we elucidated the effects of protective CD4^+^ T cells on such early inhibition of Ag- specific CD8^+^ T cell response following genital chlamydial challenge in vaccinated animals.

## Materials and methods

**Figure 1.**
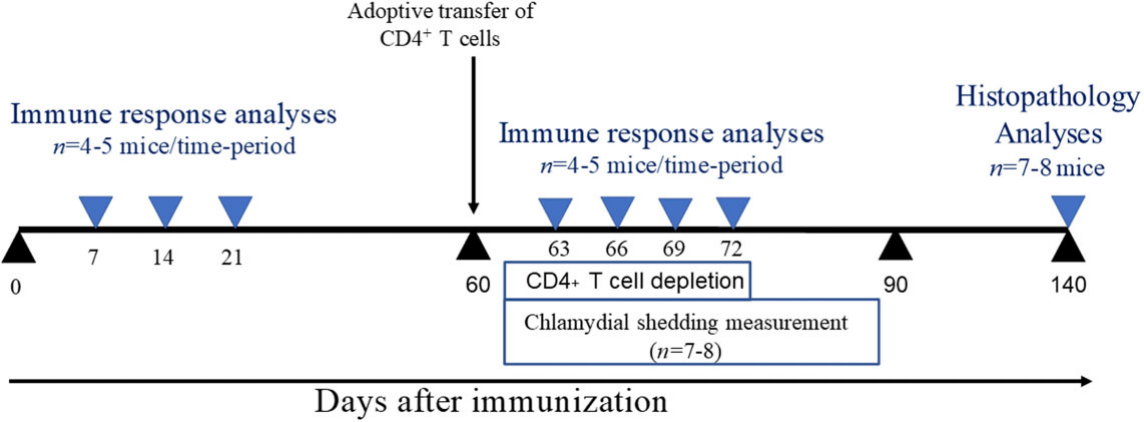
Experimental scheme. This diagram shows the various interventions and analyses conducted in this manuscript, along with the number of mice included in each experiment.

An overview of the experimental scheme used in this manuscript is shown in Fig. 1.

### *Chlamydia muridarum* and mice

*Chlamydia muridarum* Nigg (*C. muridarum*) strain was grown in HeLa 229 cells, and elementary bodies (EB) were obtained as described previously (Murthy et al. 2007). Female four- to six-week-old C57BL/6 J WT mice were purchased from the Jackson Laboratory and maintained at Midwestern University. Food and water were supplied *ad libitum* and all experiments described in this manuscript were approved by the Institutional Animal Care and Use Committee at Midwestern University.

### Immunization

Groups of mice were anesthetized (3% isoflurane) and immunized intranasally on day 0 with 2 × 10^3^ IFU EB in 20 µl of sterile phosphate buffered saline (PBS)/per mouse (live EB i.n.) or injected intramuscularly in each thigh with 5 × 10^6^ IFU EB in 0.1 ml PBS (with 0.5 µg of IL-12; live EB i.m.) per mouse. The control group were immunized intranasally (i.n.) with BSA (20 µg/mouse) 20 µl of sterile PBS alone per mouse (BSA i.n.).

### Intravaginal infection of mice, monitoring of bacterial shedding, and estimation of upper reproductive tract pathology

On day 60 after immunization, mice were challenged with 5 × 10^4^ inclusion-forming units (IFU) of *C. muridarum* contained in 10 µl of sucrose-phosphate- glutamate (SPG) buffer placed into the vaginal vault. Mice were treated with 2.5 mg of Depo- provera® (Pfizer, New York, NY) per mouse subcutaneously 5 days before vaginal challenge to induce anestrous and receptive to the genital infection. The course of infection was followed by swabbing the vaginal vault on the indicated days following inoculation (BSA i.n., *n* = 7; live EB i.m; *n* = 8, and live EB i.n., *n* = 8). Chlamydial counts were determined separately for each mouse as described previously (Murthy et al. 2007). Experiments were repeated twice and results from individual experiments analyzed independently.

The upper genital tract pathology in mice infected with *C. muridarum* was also evaluated on day 80 post-challenge, as described previously (Murthy et al. 2011). Groups of mice were euthanized, and genital tracts removed (BSA i.n., *n* = 14; live EB i.m., *n* = 15, and live EB i.n., *n* = 17) for pathology analysis. Results from two experiments were pooled.

### Splenic and draining lymph node cellular antigen (ag)-specific cytokine response

On days 7, 14, or 21 following immunizations, groups (*n* = 4–5) of mice were euthanized, draining iliac or mediastinal lymph nodes or spleens isolated, and single cell suspensions prepared. Lymph node cells (1 × 10^5^ cells/well) or splenocytes (1 × 10^6^ cells/well) were stimulated with ultraviolet (UV) irradiated chlamydial EB (multiplicity of infection, MOI = 1), or the unrelated protein bovine serum albumin (BSA), or medium alone, and incubated for 72 hours, and supernatants were collected. Supernatants from different time-periods were kept frozen at -800C until ELISA analyses. All supernatants were assayed (four replicates per *in vitro* stimulation condition) for mouse IFN-γ, TNF-α and IL-17 using Ebioscience ELISA kits (San Diego, CA), according to manufacturer's instructions.

### Splenic *chlamydia*-specific CD8^+^ T cell cytokine response after immunization or chlamydial genital challenge in immunized animals

On day 7, 14, or 21 following immunizations, or on day 3, 6, 14, or 21 after *C. muridarum* challenge, mice (*n* = 4–5 per time-period) were euthanized, spleens collected and pooled single cell suspensions for each group were prepared. CD8^+^ T cells were enriched using negative selection magnetic beads (Stemcell technologies, CA). Enriched CD8^+^ T cells (1 × 10^6^ cells/well, >95% pure, Supplementary Fig. S1) were cultured with equal number of mouse antigen presenting cells pre-infected with live *C. muridarum* (MOI = 1) or incubated with control antigens. At the end of 72 hr incubation, supernatants were collected to analyze cytokine production.

### CD4^+^ T cell depletion and monitoring

Groups of C57BL/6 mice (*n* = 20) were immunized with 20 µL of live EB i.n. (2 × 10^3^ IFU/mouse). At day 60 after immunization, mice challenged i.vag. with *Chlamydia* and were injected intravenously with 150 µg/mouse of anti-CD4 depleting antibodies (GK1.5 clone, Bio X Cell, Lebanon, NH) or rat IgG2b isotype control immunoglobulin (Bio X Cell) in 200 µl of sterile 1X PBS, every third day over a 15-day period. Some spleens were collected and analyzed using flow cytometry to confirm the CD4 depletion (Supplementary Fig. S2). Mice (*n* = 4–5 per time-period) were euthanized on day 9 and 12 following intravaginal challenge.

### Enrichment of CD4^+^ T cells from splenocytes and adoptive transfer

Groups of C57BL/6 (*n* = 20/group) mice were anesthetized and immunized i.n. with live EB or sterile 1X PBS (mock). On day 60 following immunization, spleens were collected and CD4^+^ T cells enriched using magnetic beads (Easysep, Stemcell Technologies, CA) as described previously (Li et al. 2013). The enriched (>95%) cells (Supplementary Fig. S3) were injected intravenously (1 × 10^6^ cells/mouse) into recipient mice in 100 µl sterile 1X PBS 2 hr after intravaginal infection with *C. muridarum*. Some mice which received carboxy-fluoro- succinimidyl ester (CFSE; Invitrogen by Thermo Fisher, Waltham, MA) labeled CD4^+^ T cells were euthanized on day 4 and splenic CD4^+^ CFSE^+^ cells were enumerated using flow cytometry to confirm successful transfer (Supplementary Fig. S4).

### Statistical analyses

Comparisons of two groups was conducted using Student's *t* test, whereas analysis of variance (One-way ANOVA; Systat, CA) was used for all comparisons of multiple groups. The variance between groups compared statistically was similar. The differences in incidence of oviduct pathology were compared between two groups at a time using Fisher's exact test. Linear regression analyses were conducted to determine if the level of cytokine production could predict chlamydial shedding and/or pathology as measured by the diameter of the oviduct in mice. The relationship between cytokine production after immunization or challenge to the shedding of *Chlamydia* after challenge (average of averages) or oviduct pathology (average diameter in the group) was assessed by Pearson's correlation. Differences between groups were considered statistically significant if *p* values were ≤ 0.05. All experiments were repeated at least twice, and each experiment was analyzed independently, except for upper genital tract pathology results wherein results from two experiments were pooled and analyzed.

## Results

### Platform for analysis of immune correlates of protection

**Figure 2.**
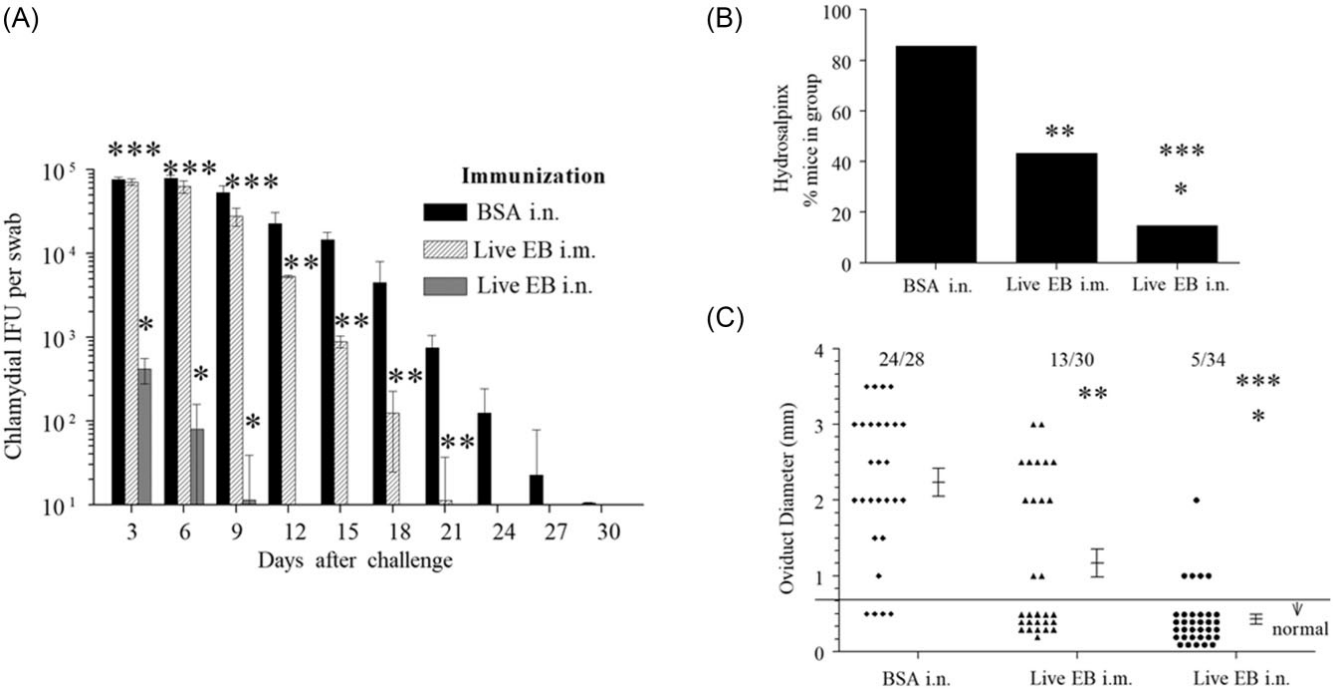
Platform for analysis of immune correlates of protection. Groups (*n* = 7–8) of C57BL/6 J mice were immunized intranasally with irrelevant antigen, BSA, or live chlamydial EB, or intramuscularly with live chlamydial EB on day 0. On day 60, mice were challenged i.vag with *C. muridarum*. (A) Mean ± SEM of vaginal chlamydial shedding at indicated time-periods is shown. Significant (*P* ≤ 0.05; ANOVA) difference between * live EB i.n. versus BSA i.n., ** live EB i.m. versus BSA i.n., *** i.n live EB versus i.m. live EB. On day 80 following challenge, oviduct pathology was evaluated. (B) Percentage of mice displaying hydrosalpinx is shown. Significant (*P* ≤ 0.05; Fisher's exact test) difference between * live EB i.n. versus BSA i.n., ** live EB i.m. versus BSA i.n., *** i.n live EB versus i.m. live EB. (C) the incidence of dilated oviducts and mean ± SEM of oviduct diameter in each group is shown. Each marker represents an individual oviduct. The horizontal line distinguishes normal from dilated oviducts. The number of dilated oviducts (numerator) and total number of oviducts examined (denominator) for each group is also shown. Significant (*P* ≤ 0.05; Fisher's exact test) difference between * live EB i.n. versus BSA i.n., ** live EB i.m. versus BSA i.n., *** i.n live EB versus i.m. live EB. All experiments were repeated twice, and composite results are shown.

We compared the protective efficacy of three vaccine regimens against vaginal chlamydial shedding and oviduct pathology. Mice immunized with BSA i.n. displayed high titers of vaginal chlamydial shedding early, followed by a progressive reduction and cessation of shedding by day 30 after challenge (Fig. 2A). Live *Chlamydia* elementary body (EB) i.n. immunization induced dramatic reduction in shedding as early as day 3 after challenge, and induced cessation of shedding by day 12 after challenge, as shown previously (Li et al. 2010). Live EB i.m. immunization displayed comparable vaginal chlamydial shedding to BSA i.n. until day 9, followed by significant reduction in shedding from days 12 to 21, and cessation of shedding by day 24 after challenge. We also measured the incidence (Fig. 2B) and severity (Fig. 2C) of oviduct pathology on day 80 after challenge. We found that live EB i.n. immunization induced near-total protection, live EB i.m. immunization induced 50% protection, and irrelevant antigen (BSA i.n.) immunized animals displayed no protection against oviduct pathology following i.vag. *C. muridarum* challenge (Fig. 2A). Furthermore, the severity of oviduct pathology was significantly reduced in live EB i.n. and live EB i.m. immunized mice, when compared to BSA i.n. immunized animals. These results were expected as we and others have previously demonstrated the protective efficacies of these regimens individually in different experimental contexts (Murthy et al. 2011, Lu et al. 2012). These models served as a platform of near-total protection, moderate protection, and no protection against vaginal chlamydial shedding and oviduct pathology in which immune correlates were further characterized.

### Total cellular cytokine response after immunization

**Figure 3.**
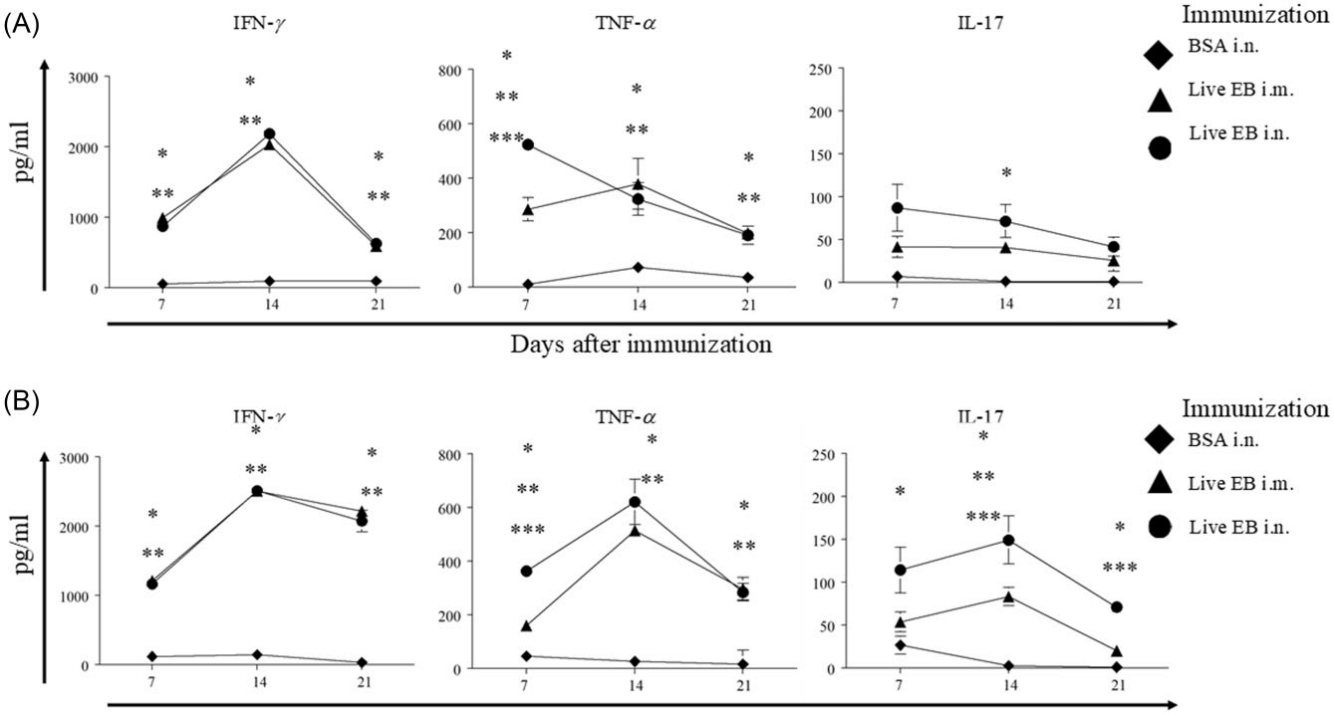
Total cellular antigen-specific cytokine response after immunization. Groups (*n =* 15) of C57BL/6 J mice were immunized intranasally with irrelevant antigen, BSA, or live chlamydial EB, or intramuscularly with live chlamydial EB on day 0. On day 7, 14, or 21 after immunization, mice (*n* = 4–5) were euthanized. Lymphocytes from draining iliac or mediastinal lymph nodes (A) or splenocytes (B) were stimulated *in vitro* with UV-irradiated chlamydial EB. Mean ± SEM of IFN-γ, TNF-α, or IL-17 production is shown. Significant (*P* ≤ 0.05; ANOVA) difference between * live EB i.n. versus BSA i.n., ** live EB i.m. versus BSA i.n., *** i.n live EB versus i.m. live EB. Results from individual experiments are shown and were analyzed independently. All experiments were repeated twice to confirm the findings.

Cellular IFN-γ, TNF-α, and IL-17 response after immunization was analyzed on day 7, 14, and 21 after initial immunization in single cell suspensions of spleen and draining lymph nodes (DLN). As shown in Fig. 3A, live EB i.n. immunization and live EB i.m. immunization both induced robust Ag-specific IFN-γ, TNF-α, and IL-17 in DLN cells on day 7, 14 and 21 after immunization. A similar response was also found in the splenocytes (Fig. 3B). Splenocytes and draining lymph node cells from BSA i.n. immunized animals displayed minimal Ag (*Chlamydia*)-specific cytokine response. These results demonstrated that an antigen-specific cellular immune response was induced as expected in the three groups of immunized animals.

### Splenic CD8^+^ T cell response after immunization

**Figure 4.**
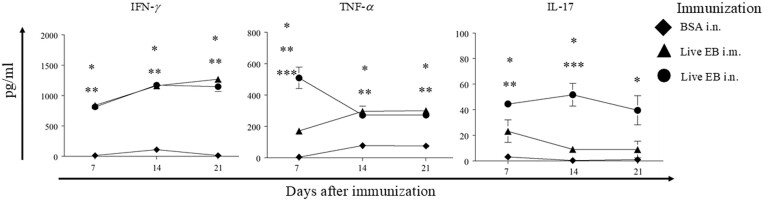
Splenic CD8^+^ T cell- antigen-specific cytokine response after immunization. Groups (*n* = 15) of C57BL/6 J mice were immunized intranasally with irrelevant antigen, BSA, or live chlamydial EB, or intramuscularly with live chlamydial EB on day 0. On day 7, 14, or 21 after immunization, mice (*n* = 4–5) were euthanized and CD8^+^ T cells were purified from splenocytes. Purified CD8^+^ T cells were stimulated *in vitro* with live chlamydial EB-infected antigen presenting cells. Mean ± SEM of IFN-γ, TNF-α, or IL-17 production is shown. Significant (*P* ≤ 0.05; ANOVA) difference between * live EB i.n. versus BSA i.n., ** live EB i.m. versus BSA i.n., *** i.n live EB versus i.m. live EB. Results from individual experiments are shown and were analyzed independently. All experiments were repeated twice to confirm the findings.

We further evaluated the splenic *Chlamydia*- specific IFN-γ, TNF-α, and IL-17 CD8^+^ T cell response in the three groups of animals on day 7, 14, and 21 following immunization (Fig. 4). As expected, the response in BSA i.n. immunized mice was minimal. Splenic CD8^+^ T cells from live EB i.n. and live EB i.m. immunized mice displayed robust *Chlamydia*-specific IFN-γ, TNF-α, and IL-17 response at all evaluated time- periods. Live EB i.n. immunized mice displayed significantly enhanced CD8^+^ T cell TNF-α response on day 7 and IL-17 response on day 14 after immunization. However, Pearson co- efficient did not indicate a significant correlation between these elevated responses and either enhancement of vaginal chlamydial shedding or reduction of oviduct pathology. Therefore, the measured Ag-specific CD8^+^ T cell responses after immunization were not reflective of the protective capability, or lack thereof, of the evaluated anti-chlamydial vaccine regimens.

### Splenic CD8^+^ T cell response following immunization and vaginal chlamydial challenge

**Figure 5.**
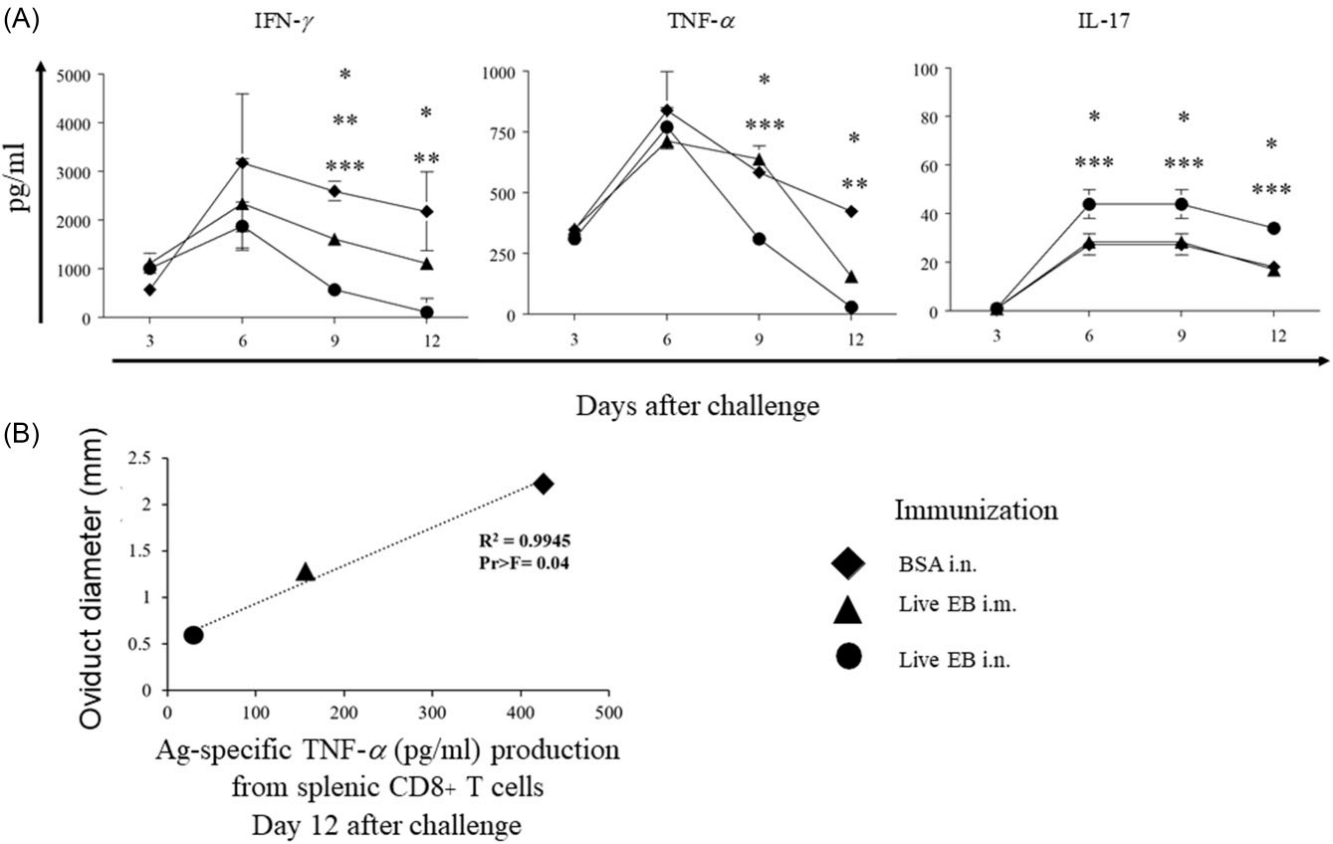
Splenic CD8^+^ T cell- antigen-specific cytokine response after challenge. A. Groups (*n* = 20) of C57BL/6 J mice were immunized intranasally with irrelevant antigen, BSA, or live chlamydial EB, or intramuscularly with live chlamydial EB on day 0. On day 60, mice were challenged i.vag. with *C. muridarum*. On day 3, 6, 9, or 12 after challenge, mice (*n* = 4–5) were euthanized and CD8^+^ T cells were purified from splenocytes. Purified CD8^+^ T cells were stimulated *in vitro* with live chlamydial EB-infected antigen presenting cells. Mean ± SEM of IFN-γ, TNF-α, or IL-17 production is shown. Significant (*P* ≤ 0.05; ANOVA) difference between * live EB i.n. versus BSA i.n., ** live EB i.m. versus BSA i.n., *** i.n live EB versus i.m. live EB. Results from individual experiments are shown and were analyzed independently. B. Pearson's correlation co-efficient between oviduct diameter to Ag-specific TNF-α production from splenic CD8^+^ T cells was analyzed. All experiments were repeated twice to confirm the findings.

The correlation of protective immunity to splenic Ag-specific CD8^+^ T cell responses at day 3, 6, 9, or 12 following intravaginal challenge was evaluated in the three groups of immunized animals. As shown in Fig. 5A, BSA i.n. immunized and *Chlamydia*-challenged mice displayed minimal Ag- specific CD8 T cell IFN-γ response on day 3, peak response on day 6, and maintained robust response on days 9 and 12 after challenge. The Ag-specific CD8^+^ IFN-γ response of live EB i.n. or live EB i.m. immunized mice was significantly lower when compared to BSA i.n. on days 9 or12 after challenge. Moreover, Ag-specific CD8^+^ T cell IFN-γ response on day 9 after challenge in live EB i.n. immunized mice is significantly lower when compared to live EB i.m. immunized animals.

BSA i.n. immunized and *Chlamydia*-challenged mice displayed minimal Ag-specific CD8^+^ T cell TNF-α response on day 3, peak response on day 6, and maintained robust response on days 9 and 12 after challenge. Interestingly, the Ag-specific CD8^+^ TNF-α response attained a similar peak in live EB i.n. or i.m. immunized animals as the BSA i.n. immunized animals on day 6 after challenge. The Ag-specific CD8^+^ TNF-α response of live EB i.n. was significantly reduced compared to BSA i.n. on days 9 or 12 after challenge, with a minimal response in live EB i.n. immunized animals on day 12 after challenge. The response in live EB i.m. immunized animals was significantly reduced compared to BSA i.n. on day 12 after challenge, and live EB i.n. immunized mice displayed further significant reduction compared to live EB i.m. animals on day 9 after challenge.

BSA i.n. immunized and *Chlamydia*-challenged mice displayed minimal Ag-specific CD8^+^ T cell IL-17 response on day 3, peak response on day 6, and maintained robust response on days 9 and 12 after challenge. The IL-17 response in live EB i.m. immunized animals was comparable to BSA i.n. at all evaluated time-periods, whereas that in live EB i.n. immunized mice was significantly enhanced compared to either BSA i.n. or live EB i.m. immunized animals. Pearson's coefficient analyses indicated that only the reduction in Ag-specific CD8^+^ T cell TNF-α response on day 12 after challenge correlated significantly (r^2^ = 0.99) with reduction in oviduct pathology, (Fig. 5B). Collectively, these results suggest that Ag-specific CD8^+^ T cell TNF-α response at day 12 after vaginal chlamydial challenge correlates significantly with the protective efficacy of vaccine regimen against oviduct pathology.

### Role of CD4^+^ T cells in vaccine-induced reduction of pathogenic CD8 T cell response after challenge

**Figure 6.**
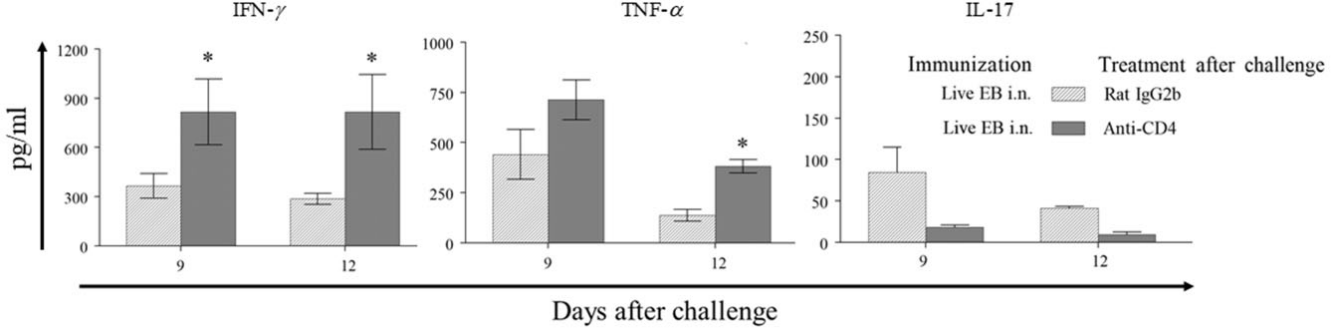
Effect of CD4^+^ T cells on splenic CD8^+^ T cell- antigen-specific cytokine response after challenge in immunized animals. Groups (*n* = 10) of C57BL/6 J mice were immunized intranasally with live chlamydial EB on day 0. On day 60, mice were challenged i.vag. with *C. muridarum* and treated with anti-CD4 antibody (*n* = 5) or rat IgG2b isotype control immunoglobulin (*n* = 5). On day 9 or 12 after the challenge, mice were euthanized and CD8^+^ T cells were purified from splenocytes. Purified CD8^+^ T cells were stimulated *in vitro* with live chlamydial EB-infected antigen presenting cells. Mean ± SEM of IFN-γ, TNF-α, or IL-17 production is shown. *Significant (*P* ≤ 0.05; Student's *t* test) difference between the groups. Results from individual experiments are shown and analyzed independently. All experiments were repeated twice to confirm the findings.

Most successful vaccine regimens described in literature rely on the induction of Th1 type Ag-specific CD4^+^ T cell response in order to induce protective immunity against genital chlamydial infection and pathology (reviewed in (Yu et al. 2016)). We hypothesized that depletion of CD4^+^ T cells after challenge would abrogate the inhibition of splenic Ag-specific CD8^+^ T cell TNF-α response at day 9 and/or 12 after challenge. Mice immunized with live EB i.n. were treated either with an anti-CD4 neutralizing/deleting antibody or with the rat IgG2b isotype control immunoglobulin after challenge. As shown in Fig. 6, Ag-specific CD8^+^ T cells from the mice depleted of CD4 T cells displayed a significant enhancement in IFN-γ response on day 9 and 10, TNF-α on day 12, and significant reduction of IL-17 on day 9 and 12 after challenge. These results demonstrate that CD4^+^ T cells are necessary to inhibit the Ag-specific CD8^+^ T cell-TNF-α response that induces pathology following genital chlamydial infection.

**Figure 7.**
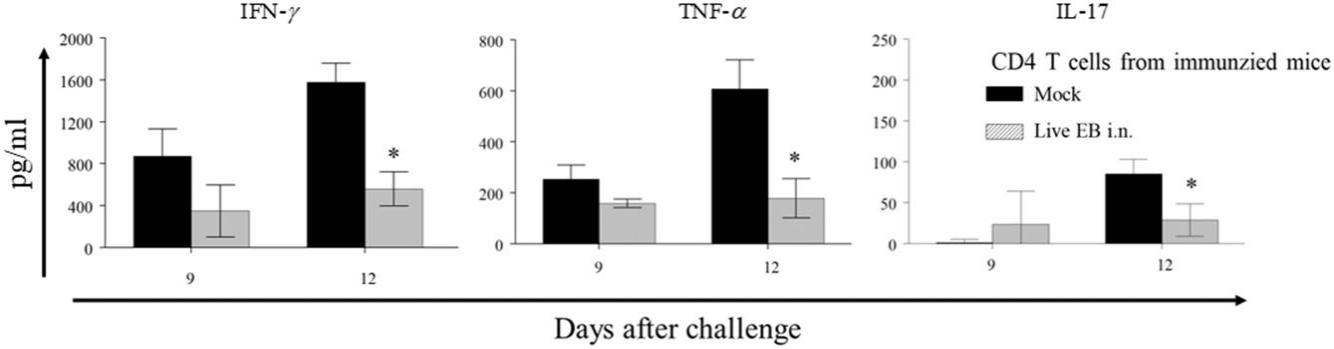
Effect of adoptively transferred *Chlamydia*-specific CD4^+^ T cells on splenic CD8^+^ T cell- Ag-specific cytokine response after challenge in recipient animals. C57BL/6 J mice were immunized intranasally with live chlamydial EB or PBS (mock) on day 0. On day 60, CD4^+^ T cells were purified and adoptively transferred (1 × 106 cells/mouse) into groups (*n* = 10) of mice challenged i.vag. with *C. muridarum*. On day 9 or 12 after the challenge, mice (*n* = 4–5) were euthanized and CD8^+^ T cells were purified from splenocytes. Purified CD8 T cells were stimulated *in vitro* with live chlamydial EB-infected antigen presenting cells. Mean ± SEM of IFN-γ, TNF-α, or IL-17 production is shown. *Significant (*P* ≤ 0.05; Student's *t* test) difference between the groups. Results from individual experiments are shown and were analyzed independently. All experiments were repeated twice to confirm the findings.

We further evaluated the sufficiency of Ag-specific CD4^+^ T cells in naïve recipient mice to mediate inhibition of Ag-specific CD8^+^ T cell-TNF-α response in mice after genital chlamydial challenge. CD4^+^ T cells from live EB i.n. immunized or mock-immunized animals were adoptively transferred into recipient unimmunized mice that were challenged i.vag. with *C. muridarum*. As shown in Fig. 7, Ag-specific CD8^+^ T cells from mice receiving Ag-specific CD4^+^ T cells displayed a significant reduction of Ag-specific CD8 T cell IFN-γ, TNF-α, and IL-17 response on day 12 after challenge, when compared to mice receiving CD4^+^ T cells from mock-immunized animals. These results suggest that Ag-specific CD4^+^ T cells induced by protective vaccine regimens are sufficient, in an otherwise untrained adaptive immune system environment, to mediate inhibition of an evolving pathogenic Ag-specific CD8^+^ T cell response following genital challenge.

## Discussion

A licensed product has yet to be attained despite several decades of research into the development of an anti-*Chlamydia* vaccine. Multiple studies using various whole or subunit vaccines have demonstrated that many regimens can successfully induce robust Th1-type CD4^+^ T cells and antibody response (reviewed in (Yu et al. 2016)). However, none induces total resistance to infection, instead a partial reduction in shedding and/or a reduction of chronic upper reproductive pathology is induced. In fact, certain candidates are successful in reducing upper reproductive tract pathology without altering the course of chlamydial shedding (Andrew et al. 2013, O'Meara et al. 2014), suggesting that in some cases vaccine-induced responses that reduce pathology may do so without affecting bacterial burden. In the past we have shown several times that TNF-α producing CD8^+^ T cell responses cause *Chlamydia*- induced upper reproductive tract pathology, likely in an antigen-specific fashion (Murthy et al. 2011, Manam et al. 2013, Manam et al. 2015, Vlcek et al. 2016). In this study, we validated the overarching hypothesis that protective CD4^+^ T cell responses induced after vaccination inhibit pathogenic CD8^+^ T cell response following genital chlamydial challenge.

CD8^+^ T cell response induces protective immunity against a number of other intracellular pathogens and such effects have been explored in *Chlamydia* infections (reviewed in (Brunham and Rey-Ladino 2005)). Despite nearly three decades of research along these lines, evidence for the role of CD8^+^ T cells in anti- chlamydial protective immunity, whether induced after natural infection or upon vaccination, is modest (reviewed in (Yu et al. 2016, Murthy et al. 2018)). One probable reason is that chlamydial organisms are largely localized to an endosomal compartment, but not in cytosol, during the developmental cycle (reviewed in (Abdelrahman and Belland 2005)). And also *Chlamydia*-infected cells have been shown to evade CD8^+^ T cell-mediated recognition and killing of infected cells (Ibana et al. 2011). Conversely, an isolated non-human primate study using the ocular trachoma model demonstrated that memory CD8^+^ T cells induced after multiple infectious challenges with a live-attenuated strain of *Chlamydia* contributed to solid protective immunity against further re-challenge (Olivares-Zavaleta et al. 2014). Certain clones of CD8^+^ T cells (Igietseme et al. 1994), especially those exhibiting specific phenotypes (Johnson et al. 2014), have been demonstrated to induce protective immunity against genital chlamydial challenge (Jiang et al. 2013, Johnson et al. 2020). One clone has been shown to possess specificity against a chlamydial protein that accesses the MHC-I presentation pathway early in the developmental cycle (Starnbach et al. 2003), possibly before the immune evasion mechanisms are induced in the infected cell. On the other hand, we have shown that depletion of the polyclonal CD8^+^ T cells results in a reduction of pathology, without affecting chlamydial clearance (Murthy et al. 2011). Moreover, repletion of TNF-α^+/+^ CD8^+^ T cells in CD8 gene deficient mice restores the pathology comparable to wild type animals (Murthy et al. 2011). Consistently, Guangming et al. (Xie et al. 2020) demonstrated that CD8^+^ T cells from OT1 mice also significantly inhibited hydrosalpinx development in wild-type mice following an intravaginal inoculation with Chlamydia. They conclude the hydrosalpinx-inhibitory CD8^+^ T cells are Chlamydia nonspecific or independent of chlamydial antigen recognition. As such, the predominant role of CD8^+^ T cells during natural chlamydial infection, at least in the mouse model, appears to be pathogenic, although certain clones and phenotypes of CD8^+^ T cells may be harnessed via vaccine platforms to induce protective immunity. Consequently, a concern was whether induction of Ag-specific CD8^+^ T cell response upon vaccination would be detrimental. Our results suggest that Ag-specific CD8 T cell responses induced upon immunization do not correlate significantly with pathological outcomes after challenge. Whereas we cannot extrapolate findings in mice directly to human individuals, our results suggest that vaccine-induced Ag- specific CD8^+^ T cell responses *per se* may not be deleterious and support the continued exploration of ways to harness vaccine-induced CD8^+^ T cell responses, or a subset thereof, to induce protective immunity against genital chlamydial infection and/or pathology.

Notwithstanding the above, we explored the correlation of vaccine-induce protection against chlamydial shedding and/or oviduct pathology to *Chlamydia*-specific CD8^+^ T cell response after genital chlamydial challenge. Interestingly, our results show that a comparable activation of peak Ag-specific CD8^+^ T cell response occurred in all groups of animals initially after challenge. However, a significant correlation of subsequent inhibition of *Chlamydia*-specific TNF-α production from CD8^+^ T cells, on day 12 after challenge, was found in animals receiving protective vaccine regimens. This suggested that the pathogenic CD8^+^ T cell program was initiated in all challenged animals but was subverted by a protective immune response quickly. The timing of such inhibition was between 6 and 12 days and correlated with the previously published timeline of 3–6 days (Roan et al. 2006, Li et al. 2008) for induction of protective CD4^+^ T cell response in challenged animals (reviewed in (Yu et al. 2016)). Furthermore, we found that depletion of CD4^+^ T cells results in enhanced Ag- specific CD8^+^ T cell TNF-α response on day 12 after challenge. Conversely, adoptively transferred CD4^+^ T cells induced a significant reduction of Ag-specific CD8^+^ T cell TNF-α response at the same time-period. In addition, the optimized OT1 mouse model study also showed that the adoptive transfer of *Chlamydia*-primed CD4^+^ T cells failed to promote chlamydial induction of pathology in OT1 mice (Zhou et al. 2022). These results demonstrate that Ag-specific CD4^+^ T cells may be necessary and sufficient, in an otherwise untrained adaptive immune environment, to quickly inhibit the pathogenic CD8^+^ T cell TNF-α response cascade, and subsequently prevent or reduce chronic upper reproductive tract pathology. The inhibition of the Ag-specific CD8^+^ T cell TNF-α response following challenge may be due to the excess pro-inflammatory cytokines including IL-12 and IFN-γ production from Ag- specific CD4^+^ T cells (Zhang and Starnbach 2015).

In summary, the results from these studies suggest that the early inhibition of *Chlamydia*-specific CD8^+^ T cell TNF-α response may be a mechanism by which protective CD4^+^ T cell responses protect against upper reproductive tract pathology following genital chlamydial infection. Furthermore, such information indicates the potential utility of Ag-specific CD8^+^ T cell TNF-α response as an early predictive biomarker of protective efficacy of vaccine regimens in future clinical trials.

## Supplementary Material

ftae008_Supplemental_Files
