# Supplementary figures and images for: Protective anti-chlamydial vaccine regimen-induced CD4+ T cell response mediates early inhibition of pathogenic CD8+ T cell response following genital challenge
Source: Pathog Dis. 2024 Apr 29;82:ftae008. doi: 10.1093/femspd/ftae008 (PMC11149721; doi:10.1093/femspd/ftae008)

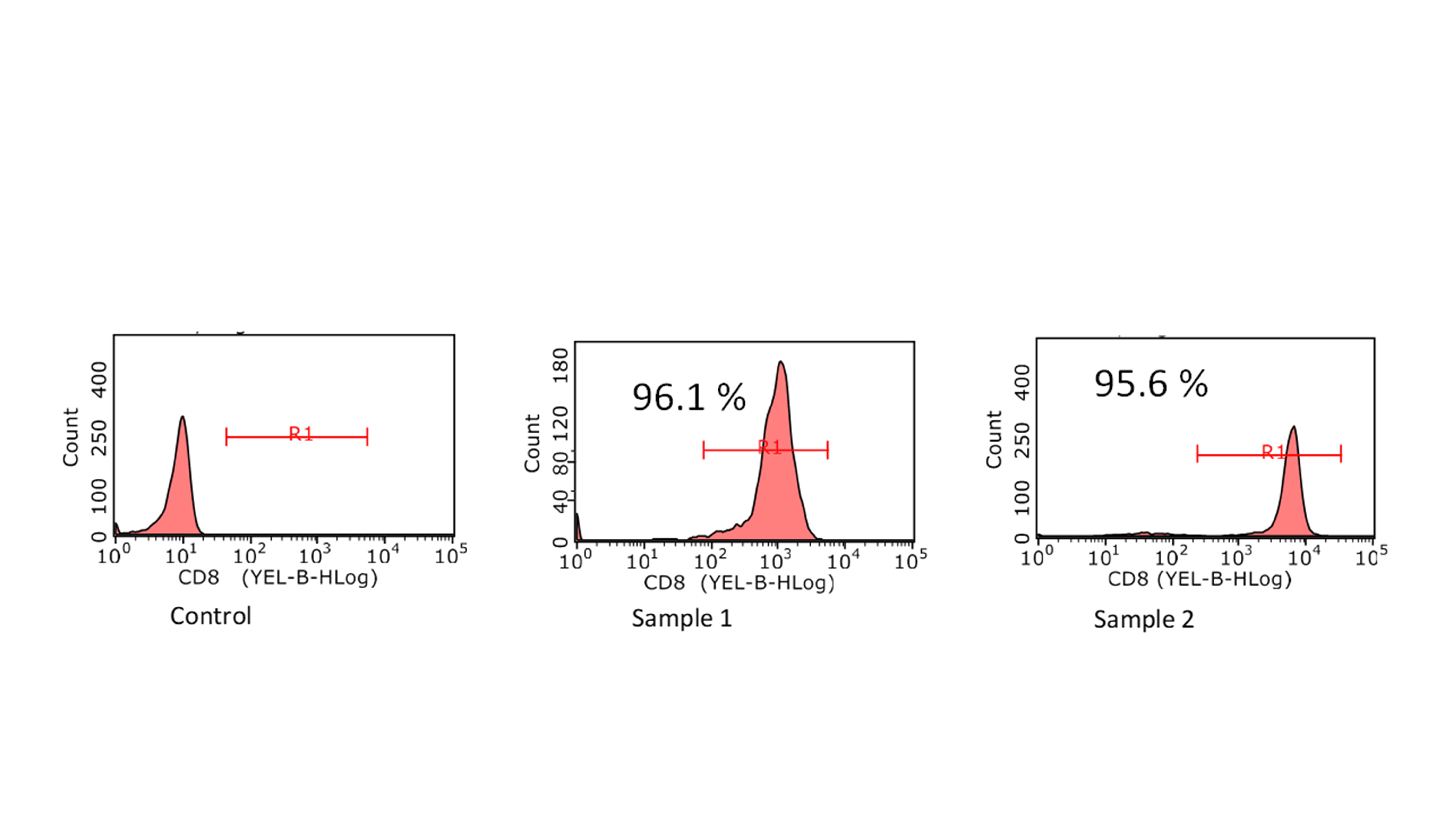

Supplement: ftae008_Supplemental_Files [file ftae008_supplemental_files.zip › Supplemental Material 1 .jpg]

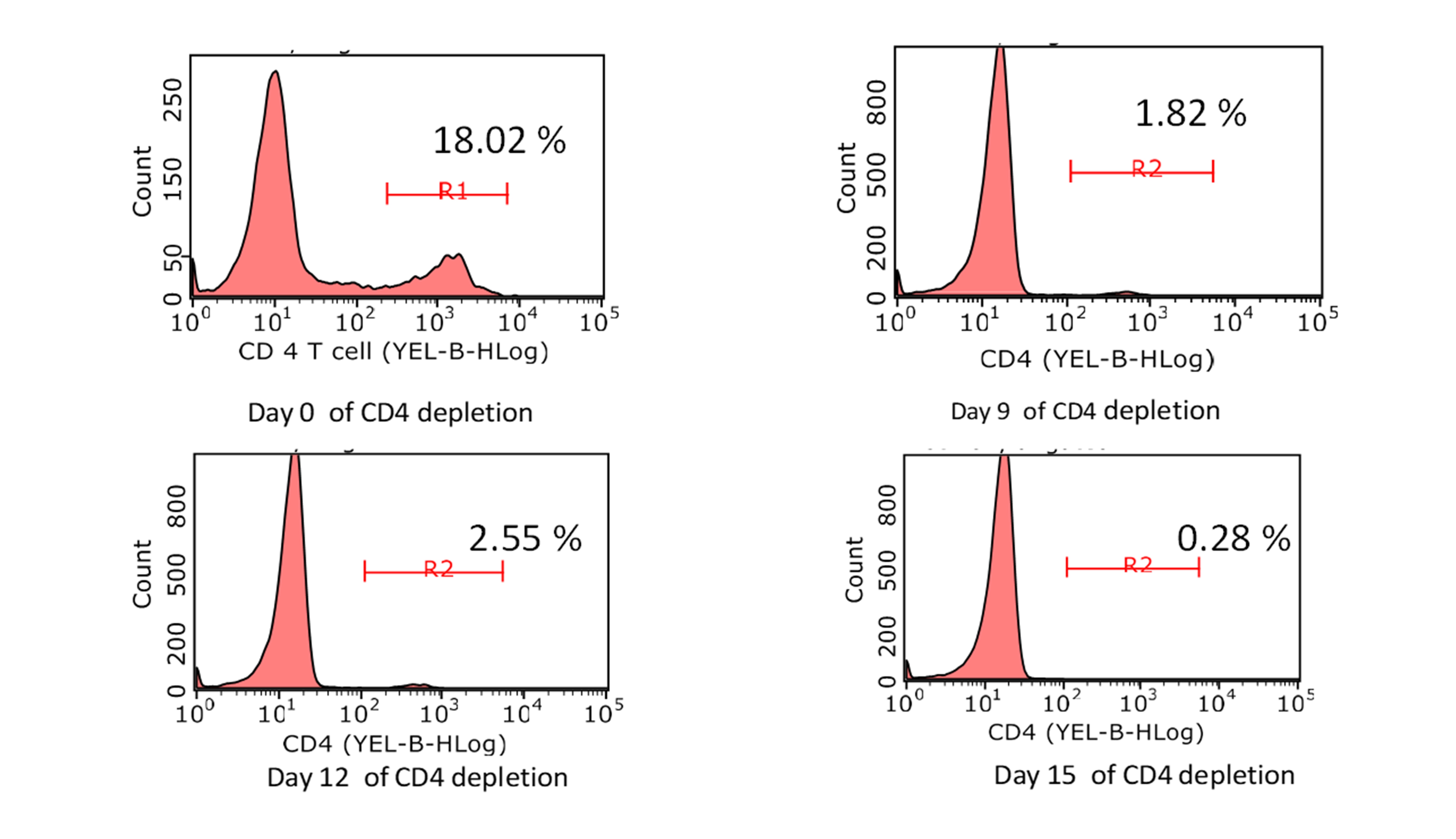

Supplement: ftae008_Supplemental_Files [file ftae008_supplemental_files.zip › Supplemental Material 2.jpg]

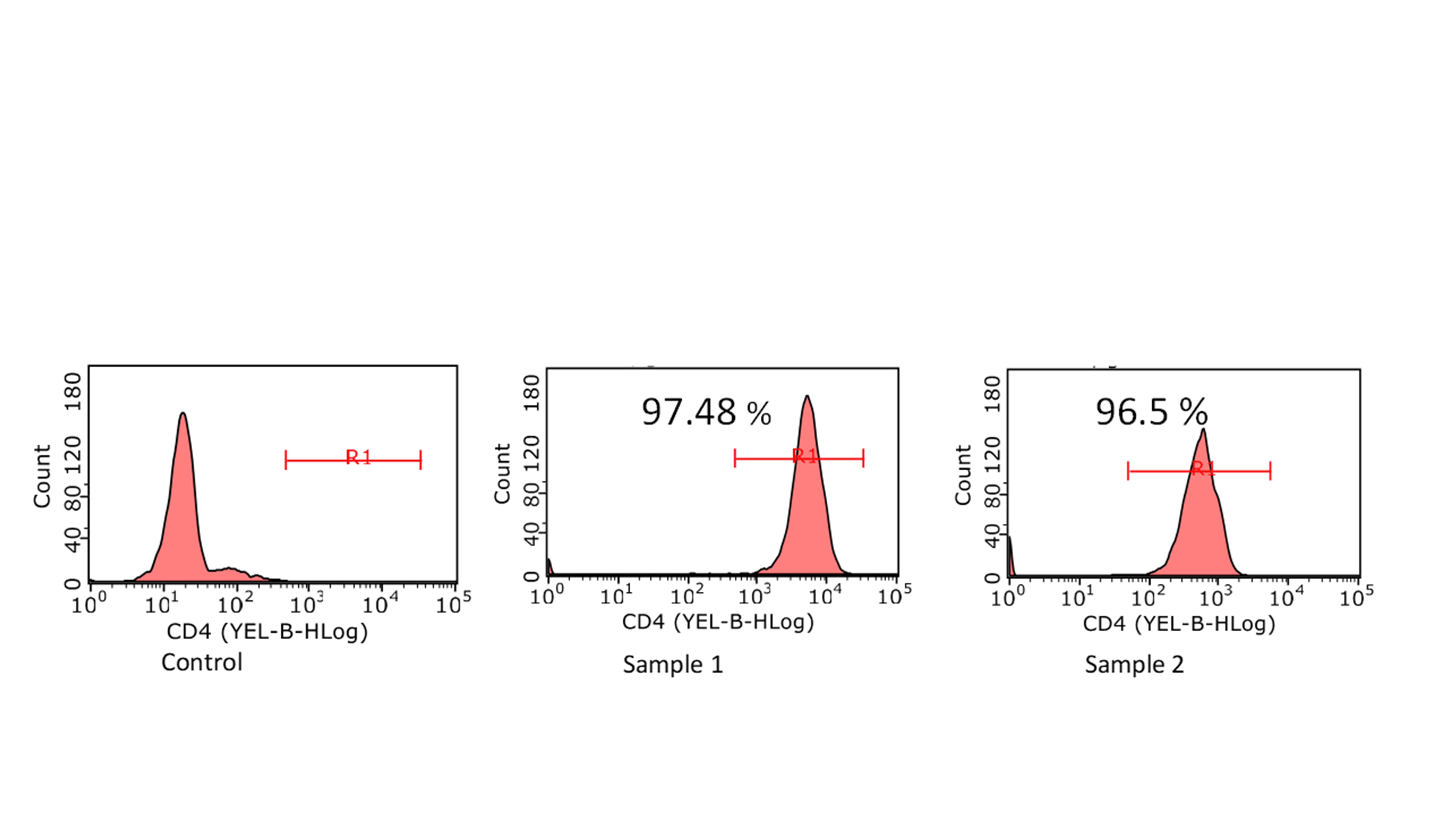

Supplement: ftae008_Supplemental_Files [file ftae008_supplemental_files.zip › Supplemental Material 3.jpg]

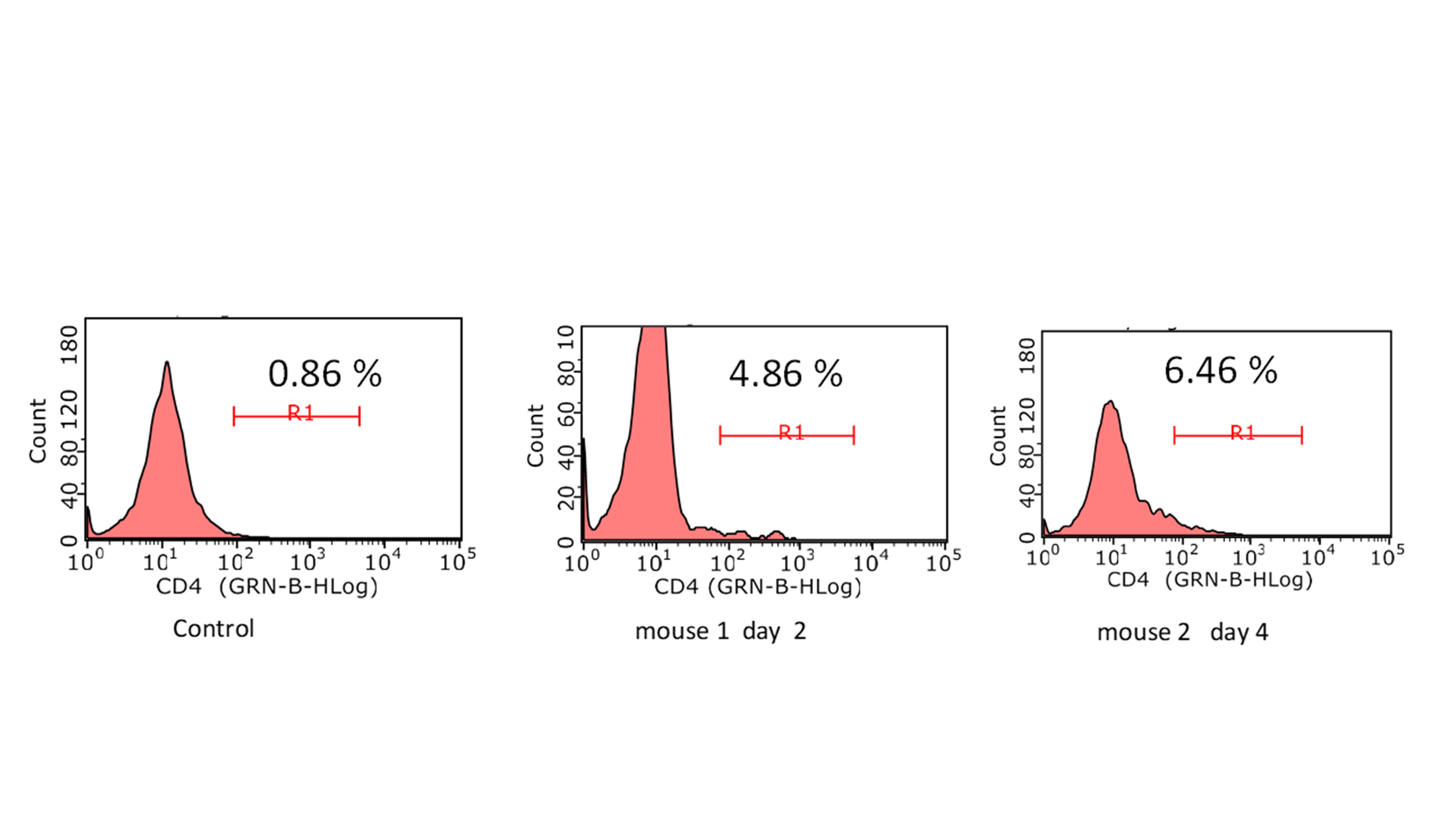

Supplement: ftae008_Supplemental_Files [file ftae008_supplemental_files.zip › Supplemental Material 4.jpg]
